# Complete Genome Sequence of Brevundimonas sp. Strain SGAir0440, Isolated from Indoor Air in Singapore

**DOI:** 10.1128/MRA.00594-19

**Published:** 2019-08-01

**Authors:** Rikky W. Purbojati, Daniela I. Drautz-Moses, Akira Uchida, Anthony Wong, Megan E. Clare, Kavita K. Kushwaha, Alexander Putra, Balakrishnan N. V. Premkrishnan, Cassie E. Heinle, Ngu War Aung, Vineeth Kodengil Vettath, Ana Carolina M. Junqueira, Stephan C. Schuster

**Affiliations:** a Singapore Centre for Environmental Life Sciences Engineering, Nanyang Technological University, Singapore; b Departamento de Genética, Instituto de Biologia, Universidade Federal do Rio de Janeiro, Rio de Janeiro, Brazil; Loyola University Chicago

## Abstract

Brevundimonas sp. strain SGAir0440 was isolated from indoor air samples collected in Singapore. Its genome was assembled using single-molecule real-time sequencing data, resulting in one circular chromosome with a length of 3.1 Mbp. The genome consists of 3,033 protein-coding genes, 48 tRNAs, and 6 rRNA operons.

## ANNOUNCEMENT

Members of the Brevundimonas genus are nonfermenting, aerobic, Gram-negative, motile bacteria classified in the phylum Proteobacteria (1). The genus was first proposed in 1994 by Segers et al., separated from the genera Pseudomonas and Caulobacter, and its type species is Brevundimonas diminuta (2). Due to its small size (1 to 4 μm by 0.5 μm), it is used as a test organism for filtration pore size and to validate reverse-osmosis filtration devices (1). *Brevundimonas* spp. have been isolated from diverse aquatic environments, such as deep-sea sediment (3) and soil (4), and have also been isolated in clinical environments from eye, lung sputum, and urine collections (1, 5). *Brevundimonas* spp. are noted to be potentially emerging pathogens causing nosocomial infections (1, 5).

*Brevundimonas* sp. strain SGAir0440 was isolated from an indoor air sample collected in Singapore (1.345141N, 103.678947E) using the Andersen single-stage impactor (SKC, USA). The air was impacted onto a Trypticase soy agar (TSA; Becton, Dickinson, USA) plate which was then incubated at room temperature. Further isolation of colonies was carried out by axenic culturing on TSA at 30°C. A single colony was grown in lysogeny broth (Becton, Dickinson) overnight before genomic DNA extraction. Genomic DNA was isolated/extracted using the Wizard genomic DNA purification kit (Promega, USA), according to the manufacturer’s protocol. Library preparation was performed with the SMRTbell template prep kit 1.0 (Pacific Biosciences, USA), followed by single-molecule real-time (SMRT) sequencing on the Pacific Biosciences RS II platform.

The PacBio sequencing run produced 88,597 subreads for a total of 972,260,241 bp and with an *N*_50_ length of 15,742 bp. These reads were used for *de novo* assembly with Hierarchical Genome Assembly Process (HGAP) version 3 (6) and polished using Quiver (6) within the PacBio SMRT Analysis 2.3.0 assembly protocol. The polished assembly was then circularized and reoriented with Circlator 1.1.4 (7), resulting in a final assembly of 1 circular contig, namely, a chromosome with a length of 3,153,531 bp (256-fold coverage). The chromosomal contig had a G+C content of 66.2%.

Taxonomic identification was performed using the full-length 16S gene sequence, extracted with Barrnap 0.7 (8), and average nucleotide identity (ANI) of the genome. The BLASTn (9) alignment of the 16S rRNA gene sequence to the NCBI nr/nt database showed 98.9% sequence identity and 99% query coverage match to the Brevundimonas vesicularis strain IAM 12105 16S rRNA gene sequence (GenBank accession number NR_112078). Average nucleotide identity analysis was conducted with OrthoANI (10) and resulted in 94.2% similarity to Brevundimonas vesicularis NBRC 12165 (NCBI assembly accession number GCF_001592205). Due to the low ANI value, this isolate was assigned to the genus *Brevundimonas* based on the 16S rRNA BLASTn results (11).

The genome was annotated with the NCBI PGAP and the RAST*tk* (12) Web server. Based on the PGAP results, 3,033 protein-coding genes (PCGs), 6 rRNA operons (2 each of 5S, 16S, and 23S rRNAs), and 48 tRNAs were predicted. In addition, the RAST annotation denotes multiple genes associated with fluoroquinolone and beta-lactam drug resistance. This also occurs in its type species Brevundimonas diminuta, which has been reported to have natural resistance to both (13).

### Data availability.

The complete genome sequence of *Brevundimonas* sp. strain SGAir0440 has been deposited in the NCBI database under the accession number CP039435, and its raw sequencing data were submitted to the NCBI SRA database under accession number SRR8894902.
